# Prediabetes: Adherence to Nutrition Visits Decreases HbA1c in Children and Adolescents

**DOI:** 10.3389/fendo.2022.916785

**Published:** 2022-06-22

**Authors:** Sadichchha Parajuli, Gabrielle Jasmin, Hannan Sirak, Austin F. Lee, Benjamin Udoka Nwosu

**Affiliations:** ^1^Division of Pediatric Endocrinology, Department of Pediatrics, University of Massachusetts Medical School, Worcester, MA, United States; ^2^Department of Population and Quantitative Health Sciences, University of Massachusetts Medical School, Worcester, MA, United States; ^3^Division of Pediatric Endocrinology, Department of Pediatrics, Zucker School of Medicine at Hofstra/Northwell, New Hyde Park, NY, United States

**Keywords:** prediabetes, type 2 diabetes, obesity, children, medical nutrition therapy

## Abstract

**Background:**

Prediabetes, the precursor of type 2 diabetes (T2D), is on the rise in the US, but the determinants of its progression are poorly characterized in youth.

**Objective:**

To determine the impact of nutrition visits, as a surrogate marker of lifestyle modification, on the trajectory of prediabetes over a 4-year period.

**Hypothesis:**

Adherence to nutrition visits could reduce BMI and lower HbA1c.

**Methods:**

A 4-year retrospective study of 108 youth with prediabetes who were recommended to receive medical nutrition therapy every 3 months following their diagnosis. Subjects were divided into 2 groups: the non-adherent group who had ≤1 nutrition visit/year, and the adherent group with ≥2 nutrition visits/year.

**Results:**

There were 46 male subjects, mean age 12.4 ± 3.6y; and 62 female subjects, mean age, 13.3 ± 3.0y, p=0.2. The adherent group (n=44, 41.5%) had higher BMI z-scores, but similar values for HbA1c, metformin use, and racial/ethnic composition compared to the non-adherent group. Overall, 18(17.0%) subjects progressed to T2D in 4y and consisted of 14(22.6%) of the 62 non-adherent subjects and 4(9.1%) of the 44 adherent subjects. The non-adherent subjects progressed to T2D at a mean duration of 25.8 ± 12.6 months while the adherent subjects progressed at a mean duration of 34.9 ± 11.8 months. The hazard ratio of progression from prediabetes to T2D for the non-adherent versus adherent group was 3.88 (95%CI 1.26-11.98, p=0.02). The results remained significant after adjusting for age, sex, race/ethnicity, BMI, and metformin use.

**Conclusion:**

Adherence to nutrition visits was associated with a 4-fold reduction in the likelihood to progress from prediabetes to T2D in US youth.

## Introduction

The rising prevalence of childhood obesity in US children has led to a parallel rise in the prevalence of prediabetes and overt type 2 diabetes (T2D) (1–3). A recent national study reported that the prevalence of prediabetes has risen significantly from 11.6% in 1999-2002 to 28.2% in 2015-2018 in US youth of ages 12-19 years (4). This sharp increase is on target to reach the adult prediabetes prevalence of 34% (3, 5).

Prediabetes has severe health consequences (6). A recent Danish population study in adult subjects reported that the highest risk for major adverse cardiovascular events (MACE) and all-cause mortality occurred in subjects with A1c levels in the prediabetic range. This study recommends increased attention to the management of cardiovascular disease (CVD) risk factors in subjects with prediabetes (6).

Prediabetes, a precursor of T2D, is well characterized in adults but less so in children and adolescents: the factors that determine the progression of prediabetes to T2D or its reversion to normoglycemia are poorly defined in the pediatric population (1, 3). Furthermore, the phenotype of prediabetes is more severe in children than in adults (2, 5) as demonstrated by a longer period of progression from prediabetes to T2D in adults of 5-10 years (5) compared to only 2-3 years in children and adolescents (2). The accelerated course of prediabetes in youth suggests an increased CVD risk in this population. However, there are insufficient data to make firm recommendations on modifiable risk factors to combat the rising trend of prediabetes in US children and adolescents (2, 4).

There is no confirmatory definition of prediabetes (1), however, the criteria for defining prediabetes by the American Diabetes Association (ADA) are based on any one of these 3 measures: impaired fasting glucose (IFG), i.e., fasting plasma glucose of 100-125 mg/dL; impaired glucose tolerance (IGT), i.e., a 2-hour post-prandial glucose of 140-199 mg/dL following a 75 g of oral glucose intake; and a hemoglobin A1c (HbA1c) level of 5.7% to 6.4% (7). The prevalence of prediabetes is variable and depends on the particular glycemic marker used for its definition (3, 8). The suitability of HbA1c as a marker for the definition of prediabetes was validated in two nationwide studies that confirmed its specificity for defining prediabetes in children and adolescents (1, 4).

There is a dearth of data on modifiable risk factors for the progression of prediabetes in youth. Data from a recent study of 2.9-year duration in youth with prediabetes found that ethnic origin was the primary determinant of reversion to either normal glucose tolerance (NGT) or progression to overt T2D. This study, however, did not explore the role of nutrition visits as all their study participants were fully adherent to nutrition visits (2). Thus, there is the need for real-world studies to identify modifiable risk factors that could impact the progression of prediabetes to T2D in children and adolescents.

We designed this study to address the poor characterization of the natural history of prediabetes in youth; and to determine the impact of adherence to nutrition visits (a surrogate marker of lifestyle modification) on the disease trajectory. We hypothesized that adherence to nutrition visits may be associated with reduced BMI and lower A1c. The study’s primary aim was to determine the frequency of progression to T2D in the first 4 years of diagnosis with prediabetes in youth. The secondary aim was to determine the impact of nutrition visits on the reversion to normoglycemia.

## Subjects and Methods

### Ethics Statement

The Institutional Review Board of the University of Massachusetts approved this study protocol under docket # H00022027. We anonymized and de-identified all subjects’ records prior to analysis.

### Subjects

In this longitudinal retrospective cohort study, we included all 108 pediatric patients of ages 4-21 years with a diagnosis of prediabetes from 2012 through 2020 who met the study’s inclusion and exclusion criteria to prevent sampling bias. All data were extracted from the Children’s Medical Center Database of the UMassMemorial Medical Center, Worcester, Massachusetts, USA. The diagnosis of prediabetes was based on HbA1c of 5.7% to 6.4% as recommended by the ADA (9) and validated in recent nationwide studies (1, 4). Subjects receiving oral anti-hypoglycemic agent, metformin, were included. Subjects were excluded if they were pregnant, incarcerated, had a history of other forms of diabetes mellitus such as type 1 diabetes and cystic fibrosis related diabetes. Subjects were further excluded if they had blood dyscrasias such as spherocytosis. Other exclusion criteria included sickle cell disease, systemic illnesses such as renal failure, liver failure, treatment with weight-loss medications such as orlistat, or other anti-hyperglycemic therapies that could impact body weight such as glucagon-like peptide-1 receptor agonists. Subjects on steroid therapy were also excluded. Eleven patients were excluded from the study based on these criteria and were not included in the analysis.

### Anthropometry

A retrospective data collection for anthropometric, clinical, and biochemical parameters was conducted on pediatric patients seen from 2012 through 2020 in our Endocrinology clinic. The methodology for anthropometry has been previously described in detail (10). Body weight was measured to the nearest 0.1 kg using an upright scale. A daily-calibrated, wall-mounted stadiometer was used to measure height to the nearest 0.1 cm. We calculated BMI from the formula: weight/height^2^ (kg/m^2^), and expressed it as z-score for sex and age, based on National Center for Health Statistics (NCHS) data (11). Overweight was defined as BMI of ≥85^th^ but <95^th^ percentile, and obesity was defined as BMI of ≥95^th^ percentile for age and gender. Pubertal status was designated by Tanner staging with pre-pubertal status as Tanner I and pubertal status as Tanner II-V.

### Assays

The methodologies of assays for laboratory chemistries have been previously described (12). Hemoglobin A1c percentage was estimated from whole blood samples and was measured using DCA 2000+ Analyzer (Bayer, Inc., Tarrytown, NY, USA) based on Diabetes Control and Complications Trial standards (13). The estimation of serum lipids was conducted at the University of Massachusetts Medical School Clinical Laboratory using the Beckman Coulter AU system based on the National Cholesterol Education Program’s criteria for accuracy (14). Serum low-density lipoprotein cholesterol (LDL-C) concentration was either measured directly by the beta quantification procedure or calculated by the Friedwald equation if serum triglycerides were ≥400 mg/dL (15).

### Medical Nutrition Therapy

MNT was provided by a registered dietician (RD), and the schedule of visits was once every 3 months following the diagnosis of prediabetes for a total of 4 visits per year **(Appendix 1)**. For this study, subjects who had ≤1 nutrition visit per year were classified as non-adherent, while those who had ≥2 nutrition visits (i.e., ≥50%) per year were classified as adherent. We chose the adherence threshold of ≥50% of the expected 4 visits per year to reflect the real-world experience of patients who may miss 1-2 nutrition visits per year for reasons other than non-adherence. The approach to nutritional assessment and recommendations focused on the intakes of macronutrients, sugary drinks, and the amount of processed food consumed by the patient. The RD educated patients on the impact of macronutrients on general health, and specifically on macronutrients and other substances that impact blood glucose levels. The RD provided further education on reading nutrition fact labels for serving size, grams of total carbohydrate, and protein. The RD made recommendations on the number of grams of total carbohydrate to be consumed based on the reduction of the carbohydrate component of the resting energy expenditure (REE) to 30-35% (16–19). In general, REE is measured in clinical practice instead of basal metabolic rate(BMR), as REE does not differ from the BMR by >10% (20). REE is estimated in a thermoneutral environment following an 8-12 hour fast (21). There are various formulae for calculating the REE such as the World Health Organization formula, the Schofield formula, and the Harris-Benedict formula (22). For this study, we used the Harris-Benedict formula (22) which has the same constants for REE calculation for children and adolescents as shown below for male and female subjects:


Male: REE = 66.47 + 13.75 × weight (kg) + 5.0 x height (cm) – 6.76 ×age (years)

Female: REE = 655.10 + 9.56 × weight (kg) + 1.85 x height (cm) – 4.68 × age (years)

Though the optimal dietary approach for weight loss is not known in children and adolescents, a reduced energy diet is known to contribute to weight loss in this population (19). The standard dietary recommendation for weight maintenance for macronutrients is 40% carbohydrates, 30 percent protein, and 30% fat, i.e., the 40-30-30 plan (16). For weight loss, using the REE formula above, it is recommended to subtract 125-250 calories per day in children < 6 years old, and 1000 calories per day in those > 6 years for a targeted weight loss of ≤2 lb per week (16). These adjustments usually reduce the carbohydrate component of the REE from 40% to 30-35% (16, 17, 19).

Protein intake, though not limited, was assessed at each visit. The RD also provided further instructions on increasing daily vegetables consumption to increase fiber and micronutrient density of meals. The RD emphasized that the recommended beverages were water, milk, and sugar-free drinks. The RD further recommended that the subjects should increase physical activity to at least 60 minutes per day. Finally, the benefits of weight loss were reviewed with the patient and internet-based apps and websites for meal planning, carbohydrate counting and activity schedule were reviewed with the patient and family.

### Statistical Methods

#### Power Analysis

*Post-hoc* power analysis was performed for the adjusted Cox’s proportional hazards survival analysis. With the following parameters: N=106, probability of event=0.17, R-square of predictors=0.04, hazard ratio=3.82, standard deviation=0.5, a two-sided test of alpha=0.05 yielded a power=0.796. We also performed a *post-hoc* power analysis based on the repeated-measure trend analysis, that is, the generalized linear model (GENMOD) presented in **Figure 2**. Given a Lag one autocorrelation of repeated measures of 0.5 and root mean square estimates of 0.3, for alpha=0.01 and 0.05 respectively, the power was >0.95 for testing the differences in the mean levels of A1c or the difference in the trends of A1c over time between the adherent and non-adherent subjects.

First, patients’ demographic and clinical characteristics were summarized as means and standard deviations for continuous variables, and N and % for categorical variables.

Secondly, survival analysis was performed on time-to-event, the diagnosis of T2D, from the time of the diagnosis of prediabetes. Kaplan-Meier survival curve for the time-to-event was generated, stratified by the frequency of nutrition visits, which was dichotomized into adherent subjects (≥2 nutrition visits, coded as 1) vs the non-adherent subjects (≤1 nutrition visit, coded as 0) during the study period. To compare hazard rates between these 2 strata, hazard ratio (HR) was calculated using an adjusted Cox proportional hazards model adjusted for age, sex, and race/ethnicity.

The time-to-event for subjects who did not develop T2D by the end of the 4th year following the diagnosis of prediabetes, or those who were lost to follow-up were censored. Thirdly, using generalized regression model (GENMOD), we traced the course of HbA1c progression during the 4-year period following the time of the diagnosis of prediabetes, and determined the risk factors associated with faster progression to T2D. Because the distribution of HbA1c values was skewed to the right we used gamma distribution with identity link function for the GENMOD analysis. The time variable for the trend analysis was the natural logarithmic transformation of the number of months since the diagnosis of prediabetes. Generalized Estimating Equations (GEE) was used to account for potential autocorrelations in repeated HbA1c measurements within subjects over time. Finally, also using GENMOD analysis, we traced the trends in BMI (in z-score), TC/HDL, and TG/HDL over two years since the time of the diagnosis of prediabetes, stratified by nutrition compliance. We did not impute missing values for variables in all analysis.

## Results

### Power Calculation

For Cox’s proportional hazards survival model, the *post-hoc* power for HR=3.82 and alpha=0.05 was 0.8. For the A1c trajectory analysis using a generalized linear model with repeated measures, the *post-hoc* power was >0.95 for both alpha=0.01 and 0.05.

### Anthropometry and Other Parameters

There were 46 (42.6%) male, and 62 (57.4%) female subjects (**Table 1**). There were no significant differences in BMI z-scores and blood pressure between the male and female subjects. HDL-cholesterol was significantly higher in the female subjects. There was neither a significant difference in baseline HbA1c between the male and female subjects (**Table 1**) nor between the adherent and non-adherent subjects (**Table 2**).

**Table 1:** Anthropometric and biochemical characteristics of the subjects stratified by sex.

Parameters	All (n=108)			Male (n=46, 42.6%)			Female (n=62, 57.4%)			p value
	n	Mean	SD	n	Mean	SD	n	Mean	SD	
Age (year)	108	12.9	3.3	46	12.4	3.6	62	13.3	3.0	0.2
Height (cm)	98	157.6	15.5	41	157.8	19.8	57	157.5	11.6	0.9
Height z-score	98	0.93	1.53	41	1.19	1.23	57	0.73	1.69	0.1
Weight (kg)	101	84.1	27.7	42	85.0	31.9	59	83.5	24.6	0.8
Weight z-score	101	2.54	0.90	42	2.83	1.02	59	2.33	0.76	**0.01**
BMI (kg/m^2^)	99	33.0	7.5	41	32.7	8.1	58	33.2	7.2	0.7
BMI z-score	99	2.27	0.68	41	2.41	0.85	58	2.17	0.52	0.1
SBP (mm Hg)	82	118.1	13.7	34	119.3	13.8	48	117.3	13.8	0.5
DBP (mm Hg)	82	74.2	8.4	34	74.9	8.5	48	73.8	8.4	0.6
TC (mg/dL)	68	160.7	31.0	36	159.9	36.0	32	161.7	24.8	0.8
LDL-C (mg/dL)	62	90.8	27.8	32	92.8	34.1	30	88.8	19.3	0.6
HDL-C (mg/dL)	67	40.8	8.3	35	38.6	7.3	32	43.3	8.8	**0.02**
TC/HDL	64	4.8	5.5	34	4.3	1.3	30	5.4	7.9	0.4
Non-HDL-C (mg/dL)	56	120.6	31.9	30	122.7	37.7	26	118.2	24.1	0.6
TG (mg/dL)	66	164.1	117.3	35	167.1	124.4	31	160.6	110.7	0.8
HbA1c (%) at baseline	105	5.9	0.2	45	5.9	0.3	60	5.9	0.2	0.7
Metformin dose (mg) baseline	28	973	554	10	725	249	18	1111	631	**0.03**
Race/Ethnicity		n	%		n	%		n	%	p
White		43	39.8		19	41.3		24	38.7	0.8 for white vs. all others
Black		22	20.4		9	19.6		13	21.0
Hispanic		31	28.7		15	32.6		16	25.8
Asian		3	2.8		1	2.2		2	3.2
Other		7	6.5		1	2.2		6	9.7
Missing		2	1.9		1	2.2		1	1.6

Age min=3.8 years, max=21 years. P-value was obtained from two sample t-test if variances were equal for p>=0.2; and from Satterthwaite test if variances were not equal for a p value of <0.2; or Chi-square test for categorical variable. Significant p values are bolded. Significant p values are bolded.

**Table 2:** Baseline comparison of anthropometric, biochemical, and therapeutic parameters between the adherent and non-adherent subjects.

Parameters	All (n=108)			Adherent (n=44, 41.5%)			Non-adherent (n=62, 58.5%)			p value
	n	Mean	SD	n	Mean	SD	n	Mean	SD	
Age (year)	108	12.9	3.3	44	12.1	3.3	62	13.4	3.2	0.051
Height (cm)	98	157.6	15.5	43	155.2	16.4	55	159.4	14.6	0.19
Height z-score	98	0.93	1.53	43	1.05	1.76	55	0.83	1.33	0.50
Weight (kg)	101	84.1	27.7	42	83.8	29.1	58	84.4	27.2	0.91
Weight z-score	101	2.54	0.90	42	2.80	0.88	58	2.35	0.89	**0.0134**
BMI (kg/m^2^)	99	33.0	7.5	43	33.7	6.9	56	32.4	8.0	0.39
BMI z-score	99	2.27	0.68	43	2.49	0.69	56	2.10	0.64	**0.0047**
SBP (mm Hg)	82	118.1	13.7	33	116.8	12.3	48	119.3	14.7	0.43
DBP (mm Hg)	82	74.2	8.4	33	75.1	6.4	48	74.1	9.1	0.58
TC (mg/dL)	68	160.7	31.0	29	156.0	33.3	37	165.1	29.8	0.25
LDL-C (mg/dL)	62	90.8	27.8	27	87.7	25.9	33	93.9	30.0	0.40
HDL-C (mg/dL)	67	40.8	8.3	28	41.8	8.4	37	39.9	8.4	0.38
TC/HDL	64	4.8	5.5	28	5.4	8.2	35	4.4	1.3	0.50
Non-HDL-C (mg/dL)	56	120.6	31.9	23	115.6	30.6	31	125.5	33.4	0.27
TG (mg/dL)	66	164.1	117.3	29	144.3	97.9	35	183.7	132.3	0.18
TG/HDL	65	4.4	3.8	28	3.5	2.7	35	5.2	4.5	0.08
HbA1c (%) at baseline	105	5.9	0.2	43	5.9	0.3	60	5.9	0.2	0.56
Metformin dose (mg)	28	973	554	16	922	514	12	1042	620	0.58
Race/Ethnicity		n	%		n	%		n	%	p value
White		42	40.38		16	37.21		26	42.62	0.77 (White vs. others)
Black		21	20.19		9	20.93		12	19.67
Asian		31	29.81		14	32.56		17	27.87
Hispanic		3	2.88		0	0		3	4.92
Other		7	6.73		4	9.3		3	4.92

Age min=3.8, max=21 years. Two subjects lacked nutrition visit information. Thus, there were 44 adherent subjects and 62 non-adherent subjects. p-value was obtained from two sample t-test if variances were equal for p>=0.2; from Satterthwaite test if variances were not equal for p<0.2; or Chi-square test for categorical variable. Significant p values are bolded.

Among those who received metformin therapy, the dose was higher in the female subjects compared to the male subjects, but was similar between the adherent and non-adherent subjects (**Table 2**). There was no significant difference in race/ethnicity when the white subjects were compared to the other groups. **Table 2** further shows the comparison of the subjects based on adherence to nutrition visits: at baseline, there were no differences in age, sex, metformin use, and lipid parameters between the 2 groups. BMI z-score was significantly greater in the adherent group compared to the non-adherent group.

### Survival Analysis for Progression to Type 2 Diabetes in the First 4 years of Disease by Nutrition Compliance

**Figure 1:**
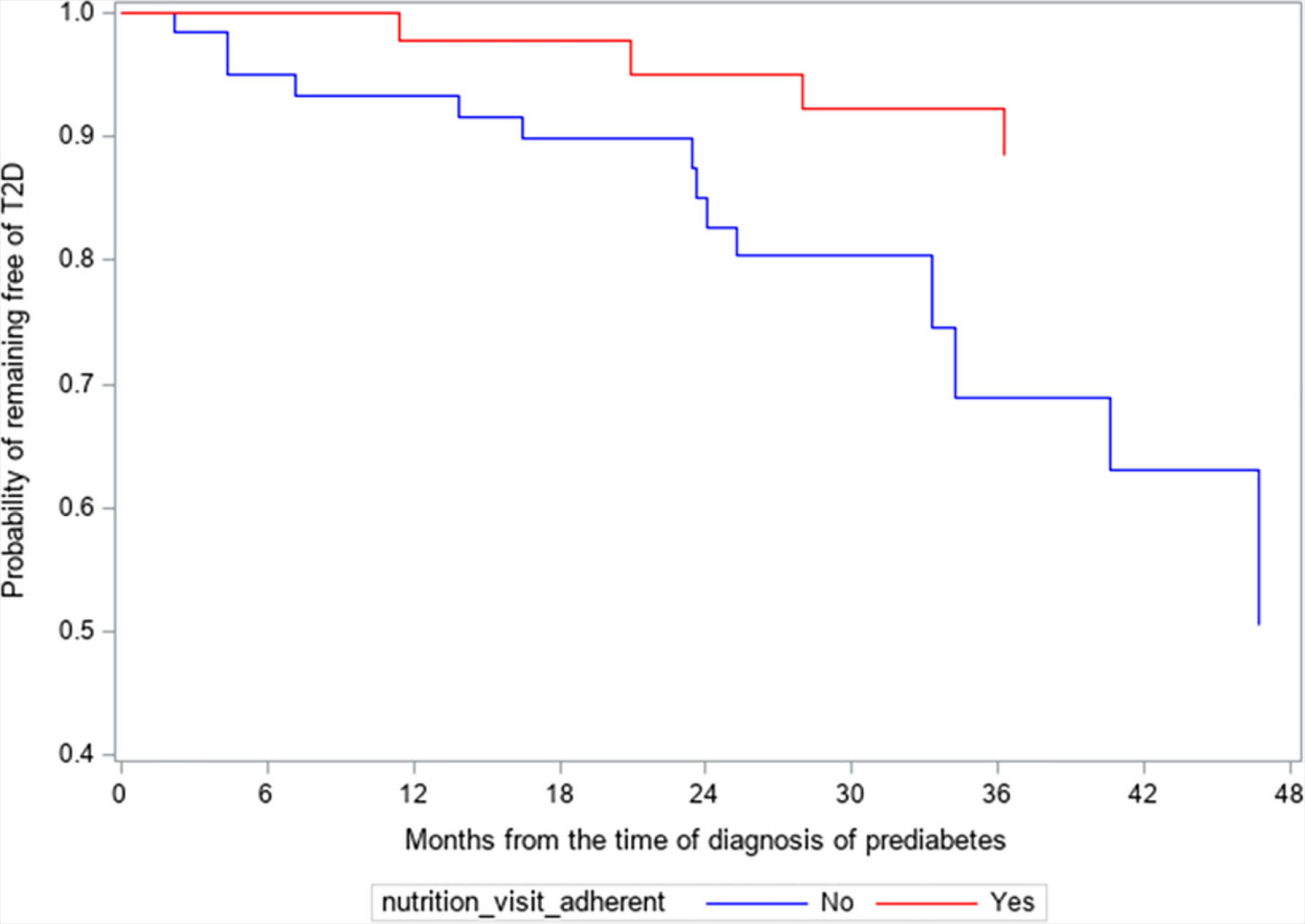
Kaplan-Meier survival analysis of the progression from prediabetes to type 2 diabetes (T2D). Hazard ratio of progressing to T2D for non-adherent subjects versus adherent subjects was 3.88 (95% CL 1.26-11.98, p=0.02).

Of the 108 subjects, two subjects were seen by an outside nutritionist. These 2 subjects did not progress to T2D in 4 years and were not included in the analysis because of difficulty with establishing their number of visits. For the remaining 106 subjects, 18 (17.0%) progressed to T2D in 4 years (**Figure 1**). Of the 62 subjects who were non-adherent to nutrition visits, 14 subjects (22.6%) progressed to T2D, with a mean progression time of 25.8 ± 12.6 months and a median of 30 months. Among the 44 subjects who were adherent to nutrition visit, 4 subjects (9.1%) progressed to T2D with a mean progression time of 34.9 ± 11.8 months and a median of 42 months. Kaplan-Meier analysis shows that the survival probability curves for progression to T2D over the 4-year study period were significantly different between the adherent and non-adherent subjects (log-rank test p=0.01) (**Figure 1**). The hazard ratio of progression to T2D from prediabetes for the non-adherent subjects versus adherent subjects was 3.88 (95% CI 1.26 to 11.98, p=0.02).

### Comparison of Changes in HbA1c Stratified by Nutrition Visits

**Figure 2:**
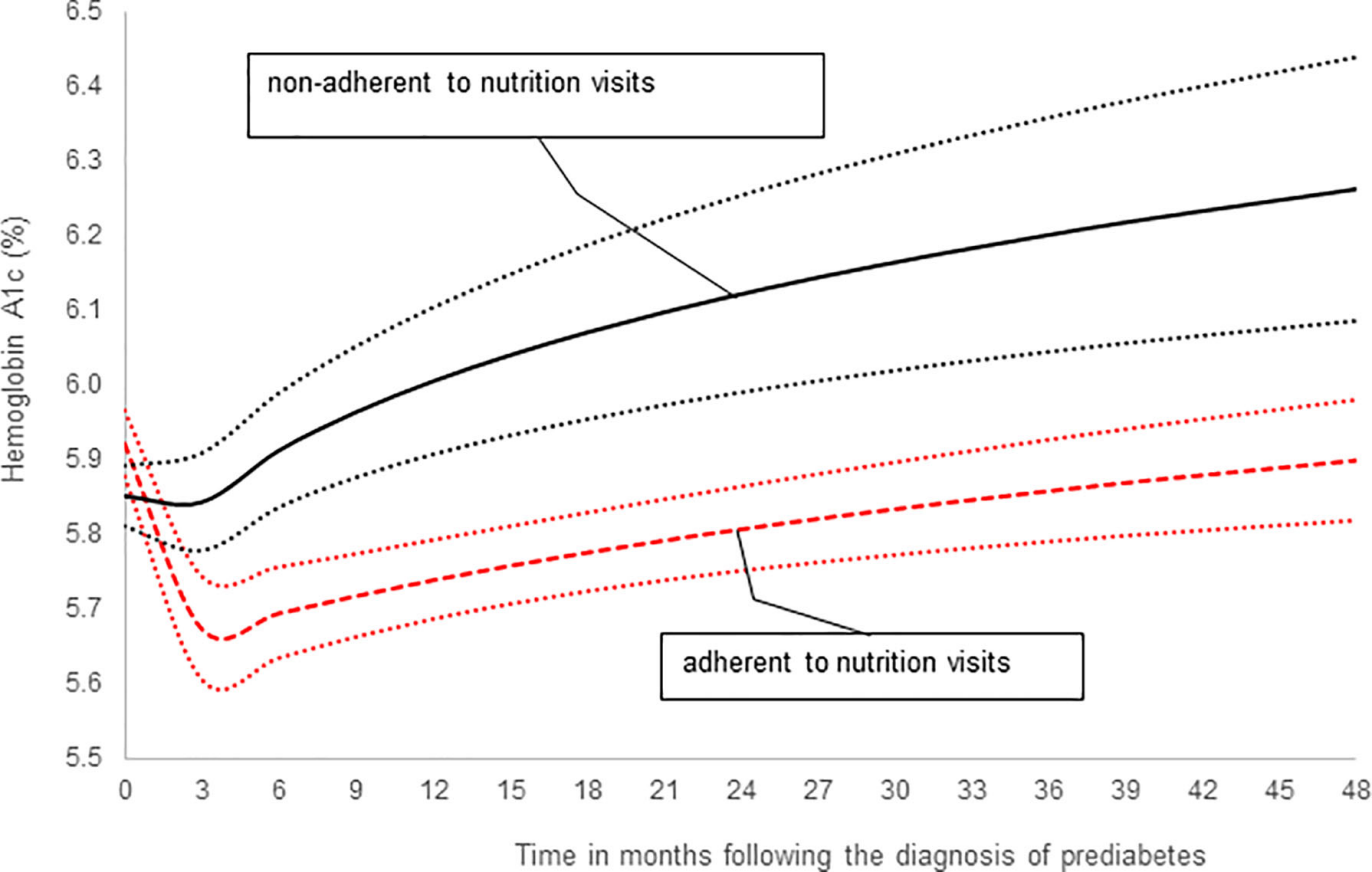
A comparison of hemoglobin A1c trends stratified by adherence to nutrition visits showing that the significant difference in HbA1c trends between the two groups persisted after adjusting for covariates: age, sex, race/ethnicity, BMI z-scores and metformin use (**Table 3**, p=0.01).

In subsequent analysis we compared the trends in HbA1c values between the adherent and non-adherent groups over 4 years (**Table 3**; **Figure 2**). **Figure 2** is a depiction of the quadratic function (in logarithmic scale) of the trend patterns for the adherent and non-adherent groups. There was a significant difference in HbA1c trend between the two groups (p=0.01, **Table 3**) even after adjusting for age, sex, race/ethnicity, BMI z-scores and metformin use. Although the adherent subjects had higher mean HbA1c value at the time of diagnosis of prediabetes, their mean HbA1c values subsequently decreased significantly compared to their non-adherent peers and stayed lower for the 4 years of study (**Figure 2**).

**Table 3:** Trend analysis of hemoglobin A1c trajectory over 48 months following the diagnosis of prediabetes.

Parameter	Estimate	SE	95% Confidence Limits		p value
**Model I: quadratic function in Ln(time) with nutritional noncompliance as classification variable**					
Intercept	5.692	0.072	5.55	5.833	<.001
Ln(months)	-0.043	0.011	-0.065	-0.02	<.001
Ln(months)*Ln(months)	0.025	0.009	0.008	0.042	0.005
Non-adherence to nutrition visits (non-adherent vs adherent)	0.093	0.067	-0.039	0.224	0.17
Ln(months)*(noncompliance)	0.070	0.028	0.016	0.124	0.01
**Model II: adjusted for age, sex, race, BMI-z score, and the use of metformin**					
Intercept	5.64	0.344	4.966	6.315	<.001
Ln(months)	-0.043	0.011	-0.065	-0.022	<.001
Ln(months)*Ln(months)	0.026	0.009	0.008	0.044	0.006
Non-adherence to nutrition visits (non-adherent vs adherent)	0.121	0.067	-0.01	0.253	0.07
Ln(months)*(noncompliance)	0.073	0.027	0.02	0.127	0.01
Age (years) at diagnosis of prediabetes	-0.006	0.019	-0.042	0.031	0.76
Sex: male vs female	0.053	0.114	-0.17	0.275	0.64
Race: white vs non-white	0.012	0.094	-0.172	0.196	0.90
Average of BMI z score over 4 years	0.038	0.069	-0.098	0.173	0.59
Use of metformin	0.006	0.096	-0.182	0.195	0.95

The trend analysis was based on generalized linear regression with gamma distribution and identity link function. Generalized Estimating Equations (GEE) was applied to account for autocorrelation within subjects over the study period of 48 months. Significant parameter estimates in Ln(months) and Ln(months)*Ln(months) indicate an overall quadratic trend in HbA1c over the study period when combining both nonadherent/adherent groups together. A significant parameter estimate in Ln(months)*noncompliance indicates difference in trends between non-adherent and adherent groups.

### Comparison of Changes in Body Mass Index, BMI Z-Score, Body Weight, Total Cholesterol/High-Density Lipoprotein Ratio, and Triglyceride/High-Density Lipoprotein Ratio Stratified by Nutrition Visits

Both TG/HDL and TC/HDL ratios have been proposed as simple markers of insulin resistance (IR) (23, 24). For example, TG/HDL ratio of 3.0 predicts IR in non-Hispanic whites while a ratio of 2.0 predicts IR in non-Hispanic Blacks (25). To explore possible mechanisms for our findings on HbA1c and nutrition visits, we investigated changes in BMI z-score, BMI, body weight; and lipoprotein fractions, TC/HDL (26) and TG/HDL (27), as surrogate marker of IR (24), between the adherent and the non-adherent groups.

**Figure 3:**
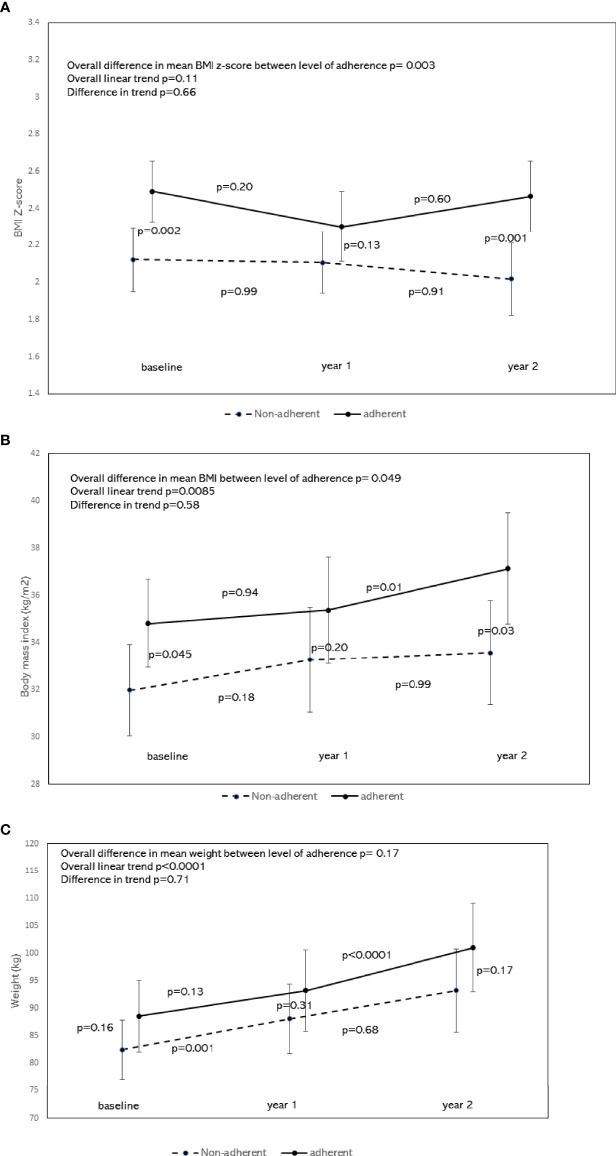
**(A)** Least square means of BMI z-score by the level of nutrition adherence with 95% confidence interval and trend analysis adjusted for age, sex, and race. To compare means of BMI z-score by nutritional adherence levels, a generalized linear model was performed on BMI z-score measured over time (baseline, year 1, and year 2). The dependent variable BMI z-score for the model was assumed to be normally distributed. Generalized Estimating Equations (GEE) was applied to account for possible autocorrelations between repeated measures. Independent variables for the model included time, nutritional adherence level (adherent vs. non-adherent), an interaction term between time and nutritional adherence level, adjusted for age, sex, and race/ethnicity. This figure shows the least-square estimated means of BMI z-score over time, stratified by nutritional adherence level with 95% confidence. There was a significant difference in the overall mean BMI z-score between the 2 groups, with the adherent group having higher BMI z-scores compared to the non-adherent group (p=0.003). Scheffé’s multiple comparisons showed that the adherent group had higher BMI z-score at baseline (p=0.002) and year 2 (p=0.001), but not at year 1 (p=0.13) when compared to the non-adherent group. There was no significant difference in the BMI z-score trend between the 2 groups (p=0.66). Scheffé’s multiple comparisons showed no changes in BMI z-score between consecutive time points at the group level: p=0.20 from baseline to year 1, and p=0.60 from year 1 to year 2 for the adherent group. Corresponding p values for the non-adherent group were 0.99 and 0.91, respectively. **(B)** Least square means of BMI by the level of nutrition adherence with 95% confidence interval and trend analysis adjusted for age, sex, and race. To compare means of BMI by nutritional adherence levels, a generalized linear model was performed on BMI measured over time (baseline, year 1, and year 2). The dependent variable BMI for the model was assumed to be normally distributed. Generalized Estimating Equations (GEE) was applied to account for possible autocorrelations between repeated measures. Independent variables for the model included time, nutritional adherence level (adherent vs. non-adherent), an interaction term between time and nutritional adherence level, adjusted for age, sex, and race/ethnicity. This figure shows the least-square estimated means of BMI over time, stratified by nutritional adherence level with 95% confidence. There was a significant difference in the overall mean BMI between the 2 groups, with the adherent group having a higher BMI compared to the non-adherent group (p=0.049). Scheffé’s multiple comparisons showed that the adherent group had higher BMI at baseline (p=0.045) and year 2 (p=0.03), but not at year 1 (p=0.20) when compared to the non-adherent group. There was no significant difference in the BMI trend between the 2 groups (p=0.58). Scheffé’s multiple comparisons for the adherent group showed no changes in BMI between baseline and year 1 (p=0.94), but a significant change between years 1 and 2 (p=0.01). There was no significant change in BMI between consecutive time points for the non-adherent group. **(C)** Least square means of weight by the level of nutrition adherence with 95% confidence interval and trend analysis adjusted for age, sex, and race. To compare means of weight by nutritional adherence levels, a generalized linear model was performed on weight measured over time (baseline, year 1, and year 2). The dependent variable weight for the model was assumed to be normally distributed. Generalized Estimating Equations (GEE) was applied to account for possible autocorrelations between repeated measures. Independent variables for the model included time, nutritional adherence level (adherent vs. non-adherent), and an interaction term between time and nutritional adherence level, adjusted for age, sex, and race/ethnicity. This figure shows the least-square estimated means of weight over time, stratified by nutritional adherence level with 95% confidence. There was no significant difference in the overall mean weight between the two groups (p=0.17). Scheffé’s multiple comparisons showed no significant difference in mean weight at each time point either. A significant change in mean weight was seen from year 1 to year 2 in the adherent group (p<0.0001), and from baseline to year 1 in the non-adherent group (p=0.001).

**Figure 3A** shows the least square estimated means of BMI z-score over time, stratified by nutritional adherence level with 95% confidence. There was a significant difference in the overall mean BMI z-score between the 2 groups, with the adherent group having higher BMI z-scores compared to the non-adherent group (p=0.003). Scheffé’s multiple comparisons showed that the adherent group had higher BMI z-score at baseline (p=0.002) and year 2 (p=0.001), but not at year 1 (p=0.13), when compared to the non-adherent group. There was no significant difference in the BMI z-score trend between 2 groups after adjusting for age, sex, and race/ethnicity (p=0.66). Scheffé’s multiple comparisons showed no changes in BMI z-score between consecutive time points at the group level: p=0.20 from baseline to year 1, and p=0.60 from year 1 to year 2 for the adherent group. Corresponding *p* values for the non-adherent group were 0.99 and 0.91, respectively.

**Figure 3B** shows the least-square estimated means of BMI over time, stratified by nutritional adherence level with 95% confidence. There was a significant difference in the overall mean BMI between the 2 groups, with the adherent group having a higher mean BMI compared to the non-adherent group (p=0.049). Scheffé’s multiple comparisons showed that the adherent group had higher mean BMI at baseline (p=0.045) and year 2 (p=0.03), but not at year 1 (p=0.20) when compared to the non-adherent group. There was no significant difference in the BMI trend between the 2 groups (p=0.58). Scheffé’s multiple comparisons for the adherent group showed no changes in mean BMI between baseline and year 1 (p=0.94), but a significant change between years 1 and 2 (p=0.01). There was no significant change in BMI between consecutive time points for the non-adherent group.

**Figure 3C** shows the least square estimated means of weight over time, stratified by nutritional adherence level with 95% confidence. There was no significant difference in the overall mean weight between two groups (p=0.17). Scheffé’s multiple comparisons showed no significant difference in mean weight at each time point either. A significant increase in mean weight was seen from year 1 to year 2 in the adherent group (p<0.0001), and from baseline to year 1 in the non-adherent group (p=0.001).

**Figure 4:**
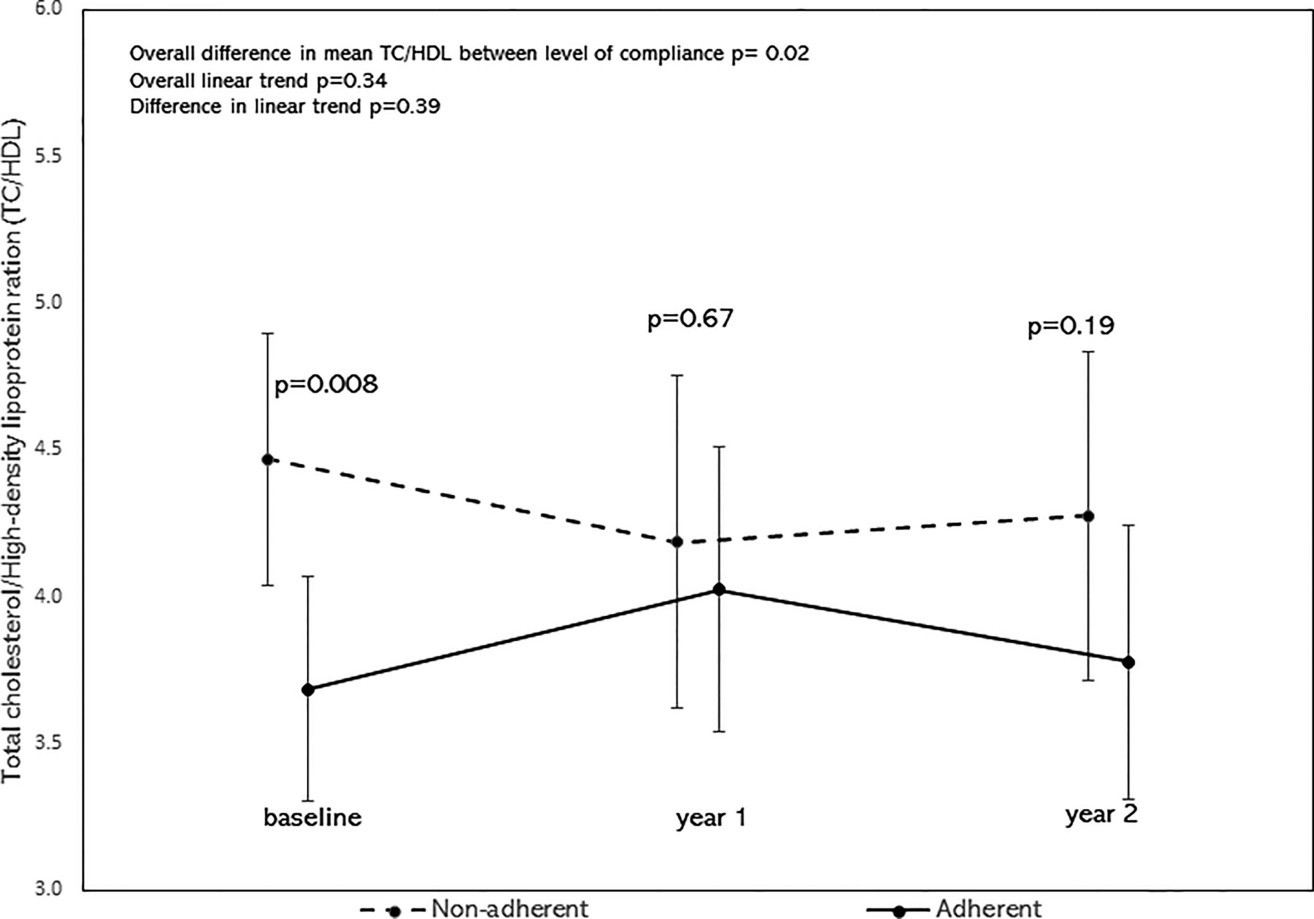
Least square means of TC/HDL stratified by adherence to nutrition visits with 95% confidence limits and trend analysis, adjusted for age, sex, race/ethnicity, and BMI-z score. The overall mean of TC/HDL was significantly lower in the adherent group (p=0.02).

**Figure 5:**
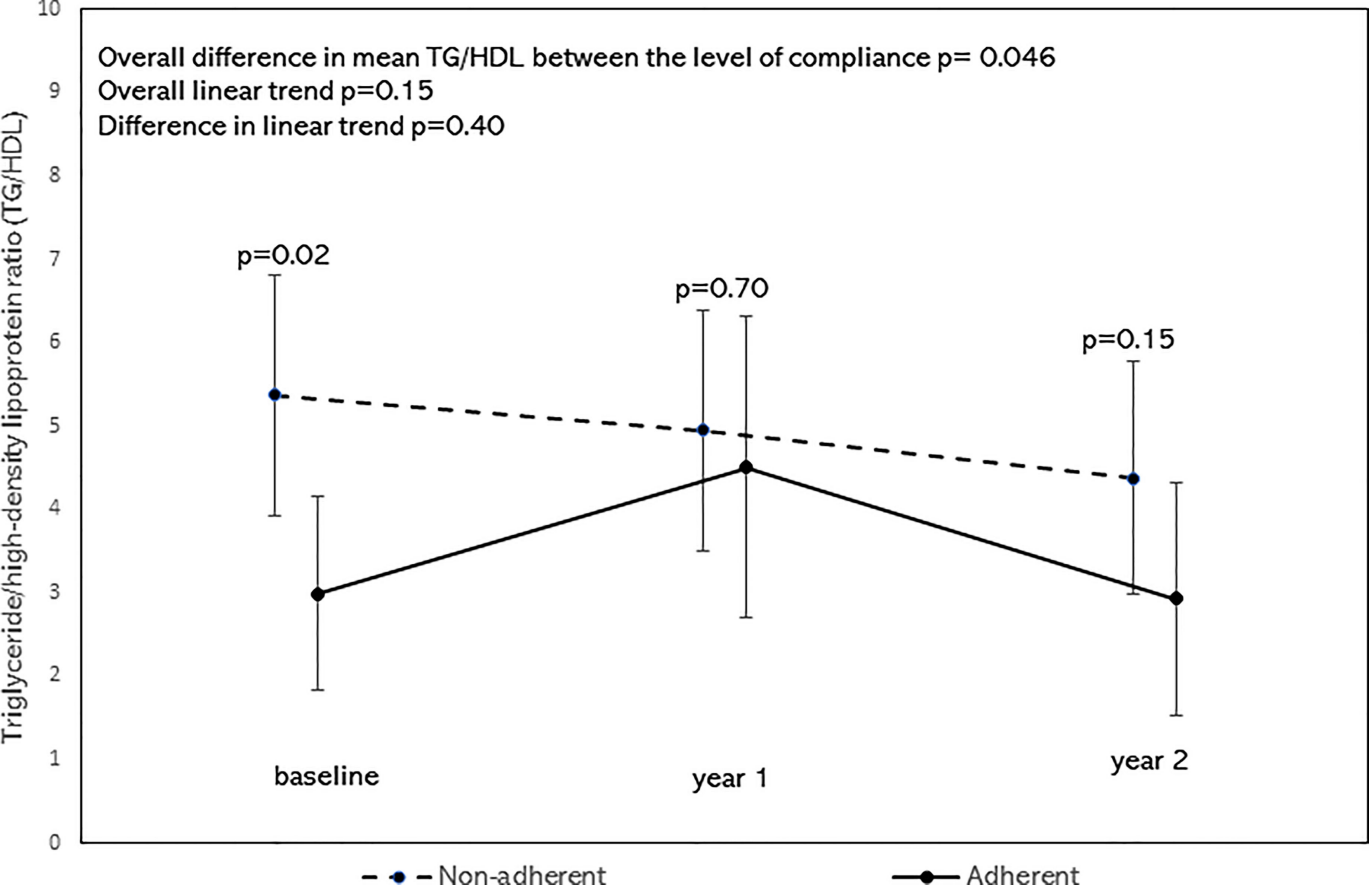
Least square means of triglyceride/high-density lipoprotein ratio (TG/HDL) stratified by Adherence to nutrition visits with 95% confidence limits and trend analysis, adjusted for age, sex, race/ethnicity, and BMI-z score. The overall mean of TG/HDL was significantly lower in the adherent group (p=0.046).

After adjusting for age, sex, BMI z-scores, race/ethnicity, the overall mean of TC/HDL was significantly lower in the adherent group as shown by the significant difference in mean TC/HDL between the adherent and non-adherent groups (p=0.02, **Figure 4**). There was no significant difference in their linear trends (p=0.39). Similarly, after adjusting for the above covariates, the overall mean of TG/HDL was significantly lower in the adherent group (p=0.046, **Figure 5**). There was no significant difference in their linear trends (p=0.40).

## Discussion

This study found that poor adherence to nutrition visits may be associated with accelerated progression of prediabetes to T2D. There was a four-fold increase in the frequency of progression to T2D in children and adolescents who were non-adherent to nutrition visits following the diagnosis of prediabetes. This finding was independent of changes in BMI z-scores, weight, or BMI (28), suggesting that phenotypic changes in anthropometric parameters may lag biochemical improvements in glycemic markers. Surrogate markers of insulin resistance (24), TC/HDL and TG/HDL, were significantly lower in the adherent subjects compared to the non-adherent subjects, suggesting that decreasing IR might contribute to the reduction in HbA1c in the adherent group.

These findings are similar to reports in adult patients with prediabetes where lifestyle modification was reported to be associated with a reduction in the rate of progression from prediabetes to T2D from 37% to 20% over a 4-year period (8). Our results differ slightly from the conclusions of a study from a pediatric obesity clinic that used impaired glucose tolerance (IGT) to define prediabetes that reported an 8% progression to overt T2D during a median follow-up period of 2.9 years (2). This study reported that ethnic origin was the primary determinant of reversion to normal glucose tolerance (NGT) or progression to overt T2D, but did not fully explore the role of nutrition visits during the study as all their subjects were adherent to medical nutrition therapy (2).

Our finding that the reversion to normal HbA1c was independent of BMI status is in agreement with a recent nationwide study that found that the increasing prevalence of prediabetes in youth is independent of obesity, race/ethnicity, and sex (4). This suggests that changes in fat mass and lean body mass may not be easily reflected by BMI z-scores. Our study is the first real-world investigation in children and adolescents to show a possible independent impact of nutrition visits on the progression of prediabetes to T2D or the reversion of prediabetes to normoglycemia over a 4-year period. We included subjects receiving metformin in this real-world study because though metformin is not approved for the management of prediabetes in children and adolescents, it is often used in this condition as an off-label prescription. Therefore, it is important to determine if metformin impacts glycemic trends following the adjustments for covariates in our findings. We saw no evidence of an impact of metformin on our results. This finding is important as it confirms the report of a randomized controlled trial by our group that showed that metformin neither impacts weight nor glycemic control in obese children with type 1 diabetes (29). It is possible that metformin has a similarr pharmacodynamic profile in prediabetes.

We speculate that the mechanisms of this change in glycemic markers in the adherent subjects are likely to be multifactorial, ranging from behavioral changes regarding improved food choices, reduced food portion size, and reduced sedentary habits. The adherent subjects were heavier than the non-adherent subjects, suggesting the possibility that youth with significant obesity were more inclined to comply with nutrition visits; or that parents of significantly obese children and adolescents were more inclined to take their children to nutrition visits. The lack of significant changes in BMI z-scores suggests that the reduction in HbA1c in the adherent subjects was not due to a reduction in BMI. It is also possible that glycemic changes precede anthropometric alterations. The significantly lower overall trend in the surrogate markers of insulin resistance, TC/HDL and TG/HDL, in the adherent group suggests that a reduction in insulin resistance may explain the decreased HbA1c in the adherent subjects.

The limitations of this study include its retrospective design which precludes any allusion to causality. Secondly, food frequency questionnaires were not administered to the subjects to assess the types and quantities of foods consumed. Third, given the retrospective nature of this study data on physical activity levels were not collected to determine the degree of adherence to the recommendations for physical activity, and the role of physical activity on the results. However, ≥50% adherence to nutrition visits seemed to correlate with a positive outcome in hemoglobin A1c.

The strengths of the study include the long duration of follow-up to establish the divergence in outcomes between the 2 groups. There was an adequate sample size to detect significant differences between the groups. We accounted for censoring in the survival analysis to allow for valid inferences. This study leveraged the advantages of HbA1c as a non-fasting glycemic marker that is specific and useful in children and adolescents (1). HbA1c level has lower within-subject variability compared to glucose (30), and does not require fasting which can be challenging in children (1).

## Conclusions

Adherence to nutrition visits was associated with reduced magnitude and speed of progression from prediabetes to T2D in US children and adolescents. There was a four-fold reduction in the likelihood of progression to T2D in youth who were adherent to nutrition visits following the diagnosis of prediabetes. This finding supports a significant role for nutrition visits, a modifiable factor, in addressing the challenge of prediabetes in youth. Larger studies are warranted to determine the mechanisms underlying improved glycemic control and reversion to normoglycemia in subjects with prediabetes who are adherent to nutrition visits.

## Data Availability Statement

The original contributions presented in the study are included in the article/**Supplementary Material**. Further inquiries can be directed to the corresponding author.

## Ethics Statement

The studies involving human participants were reviewed and approved by University of Massachusetts Institutional Review Board. Written informed consent from the participants’ legal guardian/next of kin was not required to participate in this study in accordance with the national legislation and the institutional requirements.

## Author Contributions

BN conceived the study and wrote the first draft of the manuscript. SP, GJ, HS, AL, BN contributed to the study design, data collection, analysis, and drafting of the manuscript. All authors contributed to the article and approved the submitted version.

## Funding

This study was funded in part by an investigator-initiated research grant, Grant ID: 5 R21 DK113353-03, to Benjamin U. Nwosu from NIDDK, NIH.

## Conflict of Interest

The authors declare that the research was conducted in the absence of any commercial or financial relationships that could be construed as a potential conflict of interest.

## Publisher’s Note

All claims expressed in this article are solely those of the authors and do not necessarily represent those of their affiliated organizations, or those of the publisher, the editors and the reviewers. Any product that may be evaluated in this article, or claim that may be made by its manufacturer, is not guaranteed or endorsed by the publisher.
